# The profiles of dysbiotic microbial communities

**DOI:** 10.3934/microbiol.2019.1.87

**Published:** 2019-03-21

**Authors:** Paola Brun

**Affiliations:** Department of Molecular Medicine, University of Padova, Padova, Italy

**Keywords:** gut microbiome, metagenomics, metabolomics, culturomics, bioreactors, organoids

## Abstract

Alterations in the human gut microbiota play an important role in disease pathogenesis. Although next-generation sequencing has provided observational evidence linking shifts in gut microbiota composition to alterations in the human host, underlying mechanisms remain elusive. Metabolites generated within complex microbial communities and at the crossroads with host cells may be able to explain the impact of the gut microbiome on human homeostasis. Emerging technologies including novel culturing protocols, microfluidic systems, engineered organoids, and single-cell imaging approaches are providing new perspectives from which the gut microbiome can be studied paving the way to new diagnostic markers and personalized therapeutic interventions.

## 1. Introduction

Alterations in the intestinal microbiota, termed dysbiosis, have been associated with a variety of pathological conditions including metabolic disorders, inflammatory chronic diseases, allergies, neurodegeneration, and cancer [1]–[6]. Preclinical and clinical studies have over recent decades been characterizing the changes in the composition and in the metabolic functions of the microbiota during gut dysbiosis in the attempt to expand our knowledge on the role of the gut microbiome in human diseases and to answer questions such as ‘*who's there?*’ and ‘*what genes are there?*’ [7]. It is now well established that the microbial composition can only partially explain dysbiosis-related pathologies and that the metabolic functions of the gut microbiota (‘*what's it doing?*’) also impact homeostatic and pathological conditions. Indeed, functional aspects of the microbiome could deviate from the taxonomic characterization of the microbiota, and it is possible that microbial species characterized by low gene copy numbers could contribute to gut microbiotic profiling. In the meantime, as little is known about microbial interactions in the gut and metabolic pathways under specific pathological conditions (‘*how does the gut microbiome interact with the host in a given ecosystem?*’), researchers and clinicians are continuing to struggle with the effort of translating research findings on the gut microbiome into new biomarkers for the diagnosis and management of diseases [8].

The human gut microbiota is composed of archaea, yeasts, viruses, and even some protozoans, but bacteria are, by far, the most numerous members of the community [9]. Bypassing traditional culture-dependent methods, next-generation sequencing (NGS) has allowed researchers to quantify and taxonomically classify intestinal bacteria. Metagenomics, metatranscriptomics, metaproteomics, and metabolomics together with bioinformatics and computational modelling tools are currently providing information about bacterial gene function within the complex microbial community. The large body of data that now exists has provided information on the link between dysbiosis and pathophysiological alterations, but not about microbial mechanisms of pathogenesis. To bridge this gap, experimental and clinical researchers have been exploiting multidisciplinary approaches that combine traditional microbiology and cell biology to biomedical engineering and microfluidic technology to investigate bacteria-bacteria and bacteria-host interactions.

In view of these considerations, the current work set out to briefly review the -omics technologies employed to characterize the gut microbiota dedicating particular attention to their potential and limitations. Its secondary aim was to survey the latest technical advancements such as metabolic modelling, the use of organoids and imaging approaches that may be able to explain the mechanisms underlying dysbiosis. Finally, the work provides insights into personalized therapeutic approaches based on dietary interventions, probiotic supplementation, as well as fecal microbiota or synthetic microbiota transplantation.

## 2. Sequencing technologies – ‘Who's there?’ and ‘What genes are there?’

**Figure 1.**
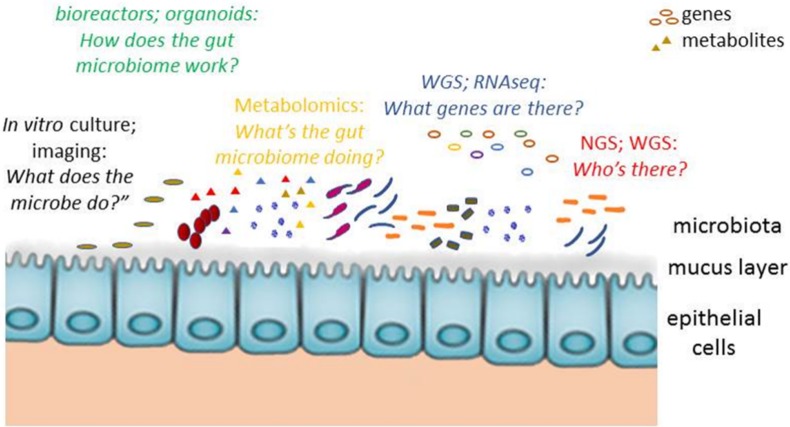
The principal approaches to studying the gut microbiota, genes and metabolites. NGS: Next-generation sequencing; WGS: whole-genome shotgun sequencing.

Bacterial gene amplicons were subjected at early research stages to restriction enzyme digestion (terminal-restriction fragment length polymorphism, T-RFLP) or separated under denaturing conditions (denaturing gradient gel electrophoresis, DGGE) or variable temperatures (temperature gradient gel electrophoresis, TGGE) to discriminate sequencing within microbial communities. Notwithstanding the fact that resolution of nucleic acids is a time-consuming technique, the method was successfully used to initially characterize the gut dysbiosis associated with pathogens or antibiotic administration and during preliminary studies investigating probiotic or prebiotic supplementation [10]–[12]. Subsequently, polymerase chain reactions (PCR)-based DNA sequencing and the next generation of massive parallel sequencing technologies (NGS) facilitated research in the gut microbiota enabling the characterization of bacterial diversity, the surveillance of microbial communities, and the detection of dysbiosis using rapid and relatively economic methodologies (Figure 1). PCR amplification of hypervariable regions such as the 16S rRNA that are shared by bacteria and archaea, the sequencing of amplicons, and bioinformatic analysis based on sequence similarity facilitate taxonomic studies and the analysis of operational taxonomic unit (OTU) abundance. It is possible to correlate microbial diversity with the diet, life style, drug assumption and pathological conditions of the host by comparing the 16S rRNA sequence profiling obtained from different experimental samples [4] even without direct data on the functional features of microbial communities. After 16S rRNA genes obtained from the fecal samples of 154 subjects were sequenced, investigators were able to identify the ‘core’ microbiota commonly found in human beings. Deviations from this core (i.e. the loss or decrease in normal microbial diversity) are associated with pathological conditions such as obesity and the metabolic syndrome [13].

NGS-based methodologies, which are currently the gold standard techniques for characterizing gut microbiota during dysbiosis, are able to detect even low concentrations of bacteria. But just as all other PCR techniques, NGSs have limitations. Sequencing hypervariable V regions of currently deposited 16S rRNA genes provides the means to identify, for example, only discrete subpopulations of the gut microbiota, thus limiting classification accuracy [14],[15]. Moreover, because bacterial genera and species have different rRNA operon copy numbers, the final annotation for NGS analysis can only infer the relative abundance of the gut microbiota which, of course, limits the possibility of uncovering subtle changes in the composition [16],[17] (Table 1).

High coverage and deep sequencing of genomic DNA, an alternative metagenomic approach provided by whole-genome shotgun sequencing (WGS approach, Figure 1), enables unrestricted sequencing of the microbial genome and generates an incredible amount of data which, when analyzed using advanced bioinformatics platforms, provides fair predictions of the taxonomic composition and the functional features [18]. Indeed, by comparing identified genes with a genomic database (i.e. the Kyoto Encyclopedia of Genes and Genomes, KEGG), it is possible to infer protein-coding sequences and the functional features of the bacterial community structure [19]. Even if the translation of identified genes to proteins does uncover some divergences [20], functional metagenomics can hypothetically unravel the mechanisms by which intestinal microbiota affects host physiology. Enzymatic pathways involved in the synthesis of short chain fatty acids are thus crucial for the homeostasis of the colonocytes in some pathological conditions such as type 2 diabetes and inflammatory bowel diseases (IBD) [21],[22]. In the same way, genes involved in the phosphotransferase system and in the metabolism of nitrate, *p*-cresol and choline are linked to obesity and ulcerative colitis [23]. Metagenomic analyses of fecal samples from 145 diabetic European females recently revealed a marked decrease in *Faecalibacterium prausnitzii* [24]. Higher levels of *Fusobacterium* and *Porphyromonas* together with disease-associated genomic markers could, for example, represent a promising non-invasive diagnostic technique in colorectal carcinoma (CRC) patients [25],[26]. Along related lines of research, the depletion of specific gut bacteria, which has been associated with markers of genetic risk, could be considered an early clinical parameter to stratify patients with IBD [27]. In addition, the WGS approach can contribute to identifying fungi, eukaryotic viruses, and bacteriophages whose role in gut microbiome-associated diseases has until now been largely overlooked. Finally, metagenomic approaches have made it possible for the International Human Microbiome Consortium to characterize the microbial genome from 300 healthy individuals generating over 14.23 terabytes of publicly available data (https://hmpdacc.org). Metatranscriptomics uses RNA sequencing (RNAseq) to characterize gene expression in the microbiome to enable microbial activity to be deciphered (Figure 1). Focusing on transcriptionally active genes, metatranscriptomics analyzes the subset of metagenomic data of only viable cells [28]. Although RNAseq offers unique insights, its limitations include RNA instability, false-sensitivity due to mRNA expression levels from organisms with higher rates of transcription, and the fact that there are no reference databases for bioinformatics analysis (Table 1). Despite these caveats, as the transcriptome varies more than metagenome, RNAseq is more accurate than the metagenomic profile and can reveal subtle microbial responses towards environmental perturbations [29]. A subject- and microorganism-specific ‘metatranscriptomic core’ of the human fecal microbiome providing a transient picture of dynamic microbial functions could potentially represent a diagnostic tool for gut dysbiosis and a feasible method for dissecting microbe-host interactions [28]. Indeed, RNAseq data has revealed that the gut microbiome modulates methylation, the expression of growth factors, ribonucleases, and cytochrome P450 activities in intestinal epithelial cells [30],[31].

**Table 1.** Advances and limitations in technologies used to study gut dysbiosis.

Technology	Applications	Potentialities	Limitations
Next generation sequencing-based methodologies	They are used to determine microbial composition	They provide taxonomic information; they detect dysbiosis	Sequenced read counts can be biased; results can be variable due to amplification of several different targeted genes
Whole-genome shotgun sequencing	It is used to determine microbial composition and the link of microbial genes with diseases	It is able to remove errors, fill in gaps or correct parts of the sequence	It cannot sequence long DNA strands and it is time-consuming
RNAseq	It is used for RNA profiling	It is used for transcriptionally active genes; it detects subtle variations	mRNA is generally unstable; there are no reference databases
Metabolomics	It is used to analyze metabolic profiles	It compares metabolites across diseases or treatments	There are no reference databases; no detection of transient metabolites
Culturomics	It is used to culture single bacterial species	It enables the growth of fastidious and unknown bacteria	Bacteria are cultured outside of their natural environment
Animal models	They are used to study microbe-host interactions	They enable the study of dynamic interactions among the gut microbiome and the host	They are expensive; gut colonization with transplanted microbes is not stable over the time
Simulators of the gut	They are used to study the dynamic growth of microbial communities	They identify the interactions among microbes (bioreactors) and host-microbe interactions (organoids) and investigate microbiome modulators	They are limited by high genetic variability and differences in immune cell composition; to avoid contaminations they require the use of antibiotics

## 3. Metabolomics – ‘What's the gut microbiome doing?’

Although metagenomics has provided important insights into gene and species composition, many functional features of the gut microbial community are still unknown. Metabolomics, which is the study of chemical processes involving metabolites, provides information about the link between gene sequencing and expression and gut microbiota activity (Figure 1). Traditional and advanced techniques such as mass spectrometry, nuclear magnetic resonance, MALDI-TOF and ion mass spectrometry offer greater accuracy and high throughput proteomic and metabolomic protocols. Integrating metabolomic data into biological systems is nevertheless a challenge (Table 1). Metabolites cannot be ascribed to a unique bacterial species since metabolic pathways are interconnected to one another and regulated by symbiosis, cross-feeding, and signals from the host [32]. *In silico* modeling of cell metabolism by means of metagenomics, metatranscriptomics and metabolomics represents a powerful, scalable tool to study the cross-modulation of metabolic pathways [33]. Genome-scale metabolic models (GEMs) were first developed to reconstruct biochemical reactions in isolated microorganisms [34], but software platforms and flux balance analysis (FBA) now make it possible to simulate the flow of metabolites through increasingly complex metabolic networks in order to map the interactions within bacterial communities and between the microbes and the host. Fang X. *et al.* combined metagenomics/metaproteomics analyses to identify enzymes degrading sugars from mucin glycan in *E. coli* B2 strains colonizing the gut of IBD patients [35]. Indeed, discrete protein modules for carbohydrate metabolism, mucin desulfation, and short-chain fatty acid (SCFA) production differentiate IBD patients and are useful in predicting the disease. A microbiome clustering is also evident in obesity. While phylogenetic studies have demonstrated an overgrowth of *Firmicutes* in obese adolescents [13], research on the gut microbiota has shown that *Bacteroidetes* are metabolically more active in the obese microbiome in producing *n*-butyrate and vitamin B12 [36],[37]. Since diet undeniably impacts gut metabolome profiling, human metabolic studies usually take into consideration subjects with similar dietary habits. Indeed, in the light of the mibrobiota's sensitivity to environmental factors and the high within and between individual variability in metabolite levels, clinical findings tend to lose their statistical significance in larger populations given the heterogeneity of microbiota derived metabolites [38],[39]. There is thus a growing demand for markers and standards that can be useful in differentiating metabolic pathways of bacteria from the host and constructing a library of microbial metabolites [40]. For example, metabolic unique functions of the gut microbiota include digestion of complex polysaccharide and fibers, synthesis of vitamins and processing of short chain fatty acids with energy production [41].

Despite these limitations, metabolomics has provided evidence for the close connection between gut microbes and the host. Indeed, metabolites of the gut microbiota affect distal body organs by modulating hormones, the immune system and brain functions [42]. The gut microbiota interprets environmental cues including drugs, pollutants and cold exposure and generates signaling metabolites such as short chain fatty acids, bile acid derivatives, and trimethylamine that impact thermogenesis or lipogenesis by binding to G-protein-coupled receptors or nuclear receptors (i.e. farnesoid receptors for bile acids) [43]. In addition, the gut microbiota catabolizes tryptophan into indole-3-aldehyde, a ligand of the aryl hydrocarbon receptor (AHR) expressed by a subset of innate lymphoid cells. AHR activation regulates intestinal mucosal homeostasis and prevents local inflammation. The gut microbiota also shapes the extra intestinal immune response in the fetus as maternal acetate, which is generated by microbial fermentation of dietary fibers, controls the immune response in the fetal lung protecting it against allergic asthma [44]. Finally, the gut microbiota synthetizes small molecules, neurotransmitters, and derivatives of indole that reach the central nervous system to regulate behavior, stress response, and to activate emotional and cognitive centers [45],[46].

But just as other -omics technologies, metabolomics has limitations which include sample collection and storage, sample processing, and the choice of database for mass spectra searching and data analysis (Table 1). Indeed, some metabolites cannot survive extraction or analysis and transient metabolites are difficult to detect because their low abundance. Metabolomics nonetheless represents the promise of individualized medicine. Genome-scale metabolic (GEM) modeling and metabolic maps applied to dysbiotic microbiota will make it possible to identify deranged metabolites and to monitor the efficacy of probiotic supplementation and microbiota replacement interventions. Similarly, GEM and flux balance analysis will make personalized synthetic gut microbiomes for transplantation a feasible possibility. Until now, fecal microbiota transplantation has been characterized by challenges such as the difficulty of finding donors, the risk of transmitting other diseases, and limited efficacy. Advances in culture of bacteria will make producing an ideal synthetic gut microbiota characterized by designed microbial cross-feeding pathways and predicted metabolites and able to improve intestinal colonization and, ultimately, to treat the dysbiosis.

## 4. Cell-based strategies – ‘What does the microbe do?’

Although the time-dependent changes occurring in the structures and the activities of the gut microbiota during the course of chronic diseases are a critical aspect of microbe-microbe and microbe-host interactions, -omics technologies are unable to guarantee real-time monitoring of gut microbiomes. Indeed, metagenomics, metatranscriptomics, and metabolomics provide a time-restricted view of the organismal complexity of the human microbiome and are unable to provide information on single microbial cells. Identifying specie-specific functions is nevertheless crucial for future clinical applications. Single-cell analysis, including cell sorting, sequencing, and cultivation will be able to fill this gap. Fluorescence-activated cell sorting (FACS) will potentially monitor single bacterial cells within a microbial community or in co-cultures of human cells [47]. Although it is difficult to have *a priori* knowledge, FACS does however need to label the cells with fluorescent probes targeting nucleic acids, proteins, or metabolites. Cell sequencing coupled to FACS sorting are able to interpret functional aspects and metabolic activities at single-cell resolution, thus overcoming the main limitation of the metagenomics approach. Protocols for amplifying genomic DNA from single cells (i.e. Multiple Displacement Amplification, MDA; Multiple Annealing and Looping-Based Amplification Cycles, MALBAC) reveal interactions between microorganisms and characterize pathogens within complex communities [48]. Although amplification biases and environmental contamination are frequently found during single-cell DNA analysis, new techniques such as microdroplet-based PCR or cross-interface emulsification guarantee precise manipulation and low contamination. Microfluidic chips can, for example, trap an individual cell in a microdroplet sorted from a complex microbial community and enable amplification and sequencing of genomic DNA in one-step procedure [49].

Functional single-cell imaging by microspectroscopy of the gut microbiota is a rapid, label-free technology that is applicable to almost all microbial species. At the moment, Raman spectra acquired from individual cells represents the most promising technique for capturing a picture of the microbial community. Raman spectroscopy interrogates the resonance frequency of the chemical bonds from the metabolites in a given cell within a population and identifies the metabolic profile of the cell, and all within a few seconds; it can trace functional changes under different environmental conditions without any labeling, isolation procedures or biomarkers [50],[51].

Microscopy and imaging technologies have led to advances in the *in situ* identification of bacteria permitting the direct observation of cellular phenotypes and functions, of their location along the gastrointestinal tract and their spatial organization in the structure of the microbial community [52]. Immunofluorescence images of DAPI-stained intestinal cross-sections and antibodies against mucin or glycans (i.e. Ulex europaeus agglutinin I, UEA-I) have uncovered higher density and diversity of the bacterial community along the longitudinal axis of the gut and spatial segregation from the host cells along the transverse axis. The biogeography of the gut microbiota localizes discrete bacterial communities in the gut lumen, mucosa and colonic crypts resulting from nutrient availability, chemical gradients, oxygen levels, and immune responses and ensures the heterogenicity in bacteria [53],[54]. Indeed, the spatial redistribution of commensal bacteria is considered the hallmark of chronic inflammatory diseases. As has been disclosed by laser capture microdissections, single cell genomics, and fluorescent in situ hybridization (FISH) to 16S rRNA, the reallocation of microbes to environments characterized by different nutrients reprograms metabolic functions and can even subvert the beneficial role of normal gut microbiota [55],[56]. Biofilms of *Bacteroides fragilis*, usually a commensal microbe in the gut microbiota, have been described in close proximity with inflamed mucosa in the biopsies of patients suffering from Crohn's disease and ulcerative colitis [57]. At the other extreme, mucin-degrading *Akkermansia muciniphila* has been implicated in wound healing when it was grown in proximity to enterocytes. In CRC, polymicrobial biofilms of commensal bacteria cluster with tumor microbiome thus revealing a previously unsuspected bacterial spatial organization [58]. Research projects using imaging techniques on transparent vertebrates such as zebrafish will enable real-time studies of host-microbe interactions tracking tumor cells and bacteria and will eventually provide new tools for diagnosing a subset of CRC [59].

A renewed interest in studying microbes in pure cultures or in defined microbial consortia to investigate the microbial species involved in human disease has recently been shown. More than ten years ago, metagenomics revealed that 80% of bacteria inhabiting the human gut were unknown and therefore unculturable [60],[61]. Information gained from metabolomics now permits environmental microbiologists to develop new approaches that are able to analyze multiple bacterial cultures. Incubation under controlled anaerobic conditions, adding supplements to growth media, efforts to detect microcolony-forming bacteria, and dilution to extinction culturing to detect minority populations have identified 70 useful conditions for culturing bacteria from freshly collected stool samples [61]–[63]. By combining different techniques (an approach referred to as culturomics), more than 50% of bacterial species previously identified by 16S rDNA have been cultured [64] and microbial species never noted before in the human gut have been isolated [65]. Culturomics enables taxonomic mapping of the gut microbiota of a donor, generating a personalized archive of bacterial clones. Even in the event *in vitro* systems should subvert the metabolic potential of isolated bacterial colonies (Table 1), a personal collection of gut bacteria could be drawn upon if dysbiosis should be identified. A culturomics + microfluidics + MALDI-TOF mass spectrometry combination enables high-throughput culturing of isolation chips (I-chips), which are multiple micro-chambers perfused with different culture media that ensure the isolation and growth of single microbial cells from a complex bacterial community [66].

## 5. Simulators of the gut microbiome – ‘How does the gut microbiome work?’

Identifying the location of discrete bacterial communities in specific area of the gut (the biogeographical distribution) that are mandatory for bacterial function have presented new challenges as far as -omics technologies are concerned. Indeed, the results of metagenomics and metabolomics analyses of stool samples or biopsies are not representative of the microbial diversity along the entire gastrointestinal tract running from the mouth to the rectum. Studies carried out on germ-free mice transplanted with only specific strains of bacteria (gnotobiotic mice) or with human fecal microbiota (human microbiota-associated mice) have provided data on microbial dynamics along the intestine and on how microbiota dysbiosis contributes to specific diseases. It is nevertheless true that animal-based research is costly and has limitations that need to be considered when results are being interpreted [67],[68]. Although only 15% of bacterial lineages are shared by humans and mice [69], nearly 85% of human microbiota can be successfully transplanted in germ-free mice. Moreover, predominant taxa fail to efficiently colonize the murine gut as they are lost over a single generation [67],[70] as the result of the selective pressure of the host immune system, diet factors and the genotype [71],[72] (Table 1).

To overcome these limitations, *in vitro* co-culture of bacteria and mammalian cells can provide a strictly controlled system with a number of possible readouts. Indeed, polarized intestinal or colonic epithelial cells grown on transwell membranes or three-dimensional scaffolds can be exposed to a microbial community seeded on the apical face [73]–[76]. Changes in transmembrane resistance, active transport, cell permeability, absorption, and excretion can be used to assess bacteria-induced dose and time dependent effects. Miniaturization of culture systems and microfluidic perfusion make it possible to isolate and characterize single bacterial cells using imaging, gene-expression profiling, or mass spectrometric analysis. The limitations of *in vitro* co-culture systems including the absence of goblet, endocrine, and immune cells led researchers to design bioreactive system-gastrointestinal tract simulators that are able to support *in vitro* growth of microbial communities under controlled conditions (i.e. temperature, pH, bile acid concentrations, nutrient gradient) and to mimic the different districts of the gut. The simulator of human intestinal microbial ecosystem (SHIME) uses a series of linked reactors reproducing the stomach, the duodenum/jejunum, the ileum/caecum and the colon. Each chamber is filled with a calibrate volume of nutritional medium, continuously stirred, and kept at 37 °C in anaerobic conditions. Inoculation with feces from healthy donors ensures colonization of each chamber of the simulator with a unique microbial community, closely resembling the ones found in the host [77]. SHIME bioreactors have been used for *in vitro* generation of microbial stool substitutes to treat *Clostridium difficile* infections [78]. In addition, mini-bioreactors were set up for high-throughput cultivation of gut bacterial communities [79] but in the absence of the host cellular component.

Gut organoids represent a valuable *in vitro* system to culture gut microbial species in proximity to host cells thus facilitating the study of host-microbe interactions. Enteroids or colonoids are spherical structures of stem cells and differentiated cells surrounding a lumen that contains mucus. In a recent work, Williamson *et al*. described a high-throughput platform of 500 colonoids with robotically controlled microinjectors to inoculate fecal-derived bacteria into the lumen. The bacterial mixture survived for more than 18 hours without significant changes in density and diversity [80]. Single microbial species or complex communities of fungi, viruses and parasites can colonize human intestinal organoids to analyze the role of microbial interactions. Intestinal organoids are, thus, powerful tools to study gut microbiome modulators since prebiotics, probiotics, bacteriophages, dietary factors and pathogenic microbes can be supplemented and monitored using imaging techniques. Moreover, by enabling the study of host cell responses, intestinal organoids can help researchers to dissect the pathways by which individual microbes exert their effects on host epithelium, as has already been reported for *C. difficile* [81]. Genome-editing systems of human intestinal organoids will provide further insights into host factors and microbial-induced pathological conditions such as IBD and cancer. It should be remembered that experiments with organoids require enough biological and technical replicates to reach statistical power as organoids report high variability in gene expression [82]. Variations in the cell lineage composition of mature intestinal organoids do not as a consequence guarantee the differentiation of immune cells or the production of antimicrobial peptides and immunoglobulins (Table 1). All these factors have an important effect on the injected microbiome and need to be considered when intestinal organoids are used to study the host-microbe interaction.

## 6. Conclusions

A growing number of studies have demonstrated that there is a link between compositional and functional alterations in the gut microbiota and disease states. Inadequate study designs and the lack of standardized methods have posed challenges to solving the riddle if alterations in the gut microbiome are *the cause or the consequence* of an altered biological state. Given the inherent complexity and heterogeneity of the gut microbiome, a multidisciplinary approach exploiting all the traditional and state of the art technologies will be needed to uncover the many secrets of the gut bacteria under different environmental conditions and to establish causal links between metabolic changes and diseases.

Given the biological variability of the gut microbiota, standardization and guidelines for best practices are, of course, mandatory. The International Human Microbiome Standard (www.microbiome-standards.org) and the Microbiome Quality Control project (www.mbqc.org) are already coordinating the development of standard operating procedures designed to optimize data quality and comparability in the human microbiome field. Standardizing human microbiome studies will facilitate designing observational and interventional studies, identifying microbial metabolites as disease biomarkers, and in customizing gut microbial communities for individualized interventions.

The simultaneous identification of all the microbial members of the microbiota and characterization of their subtle reactions to factors linked to human disease are crucial to mapping the healthy microbiome and in designing efficient therapeutic interventions. The cost-effective, high-throughput platforms and data analysis pipelines and (bio)reactor systems that are becoming available will make it possible to combine metagenomics, metabolomics, imaging techniques, and single-cell analysis to trace and scrutinize the dynamic host-microbiota interactions. Microbiome bioinformatics will be able to provide computational methods to fill the gaps of -omics technologies by integrating and comparing different experimental results. Sharing workflows for microbiome data analysis will further enhance the possibility of advancement.
